# Effect of intra-abdominal administration of ascites fluid on postoperative peritoneal adhesion in rat model: A randomized controlled trail

**DOI:** 10.1016/j.amsu.2021.102928

**Published:** 2021-10-09

**Authors:** Ali ShaykhoIslami, Mohammadreza Ghasemian, Mahmoud Zardast, Marjan Farzad

**Affiliations:** a Surgical Resident, School of Medicine, Birjand University of Medical Sciences, Birjand, Iran; b Cardiovascular Diseases Research Center, Department of Surgery, School of Medicine, Birjand University of Medical Sciences, Birjand, Iran; c Imamreza Clinical Research Development Unit, Birjand University of Medical Sciences, Birjand, Iran; d Department of Pathology, School of Medicine, Birjand University of Medical Sciences, Birjand, Iran; e Cardiovascular Diseases Research Center, School of Nursing and Midwifery, Birjand University of Medical Sciences, Birjand, Iran; f Clinical Research Development Unit of Razi Hospital, Birjand University of Medical Sciences, Birjand, Iran

**Keywords:** Surgical adhesion, Ascites fluid, Rat

## Abstract

**Introduction:**

Intra-abdominal adhesions are typically found after the most surgical procedures. Normally, most adhesions are asymptomatic; however, few individuals experience postoperative adhesion-related problems such as small bowel obstruction, pelvic pain, infertility, or other complications. We aimed to evaluate the preventive effect of the ascites fluid for postoperative peritoneal adhesions in rat models.

**Material and methods:**

This experimental trial was conducted in Sixty Syrian male rat randomly assigned to six groups of 10 animals each as follows: control (group 1&4); normal saline (group 2&5): 2 mL of normal saline was poured into the peritoneal cavity; and case (group 3&6): 2 mL ascites fluid was poured into the peritoneal cavity. All animals in the six groups underwent laparotomy and measurable serosal injury were created with a standard technique. 10 and 30 days after initial surgery, the rats underwent another laparotomy in groups 1, 2, 3 and 4, 5, 6, respectively to assess macroscopic and microscopic adhesions, which were scored by an examiner who was blind to the animals̕ group assignment. Data analyzed by SPSS version 18, using the kruskal Wallis and Bonferroni-corrected Mann-Whitney U tests. P-values of less than 0.05 were considered significant.

**Results:**

The mean scores of both microscopic and macroscopic adhesion were significantly different between all the groups (P < 0.05). Total macroscopic and microscopic adhesion scores were significantly lower in the ascites fluid treatment than in the control (P = 0.0001) or the normal saline (P < 0.001) group. There was no significant difference between adhesion intensity 10 and 30 days after laparotomy (P > 0.05).

**Conclusions:**

Ascites fluid can decrease the possibility of post-operative intraperitoneal adhesion formation.

## Highlights

• Ascites fluid decreases post-operative adhesion formation.
• The intensity of adhesion did not differ over time.
• Ideal adhesion-reducing substances are still under investigation.

Protocol Number: IR. BUMS 1398.218.

## 1 Introduction

Abdominal adhesions are typically formed after abdominal surgery. They are fibrous bands that cover two or more intra-abdominal organs or the peritoneal membrane. Adhesions also result from inflammatory conditions of the abdomen or abdominal-pelvic radiation. Normally, most adhesions are asymptomatic; however, few individuals experience post-surgery adhesion-related problems such as small bowel obstruction, pelvic pain, infertility, or other complications [1]. Cadaver studies revealed adhesions in 67% of patients with prior procedures and in 28% with a previous intra-abdominal infection. Abdominal adhesions are the most common cause of bowel obstruction (65%–75%), and lower abdominal procedures have a higher chance of producing adhesion and obstruction [2].

Adhesion formation is a major concern of all surgeons and minimizing surgical trauma is a factor to reduce adhesions. Minimizing the surgical trauma includes avoiding desiccation, gentle handling, reducing foreign body exposure, and securing hemostasis. Despite the advances in surgical techniques made for adhesion diminution, the incidence of adhesions is still high [3].

Ascites is the abnormal build-up of fluid in the abdomen. Technically, it is more than 25 ml of fluid in the peritoneal cavity. The most common cause of ascites (84%) is liver cirrhosis [4]. Ascites fluid can accumulate as a transudate or an exudate. Roughly, transudate results from increased pressure in the hepatic portal vein (>8 mm Hg, usually around 20 mm Hg *e.g.*, due to cirrhosis), while exudate is actively secreted fluid due to inflammation or malignancy. As a result, exudate is high in protein and lactate dehydrogenase and has a low pH (<7.30), a low glucose level, and more white blood cells. Transudates have low protein (<30 g/L), low LDH, high pH, normal glucose, and less than 1 white cell per 1000 mm³. Clinically, the most useful measure is the difference between ascetic and serum albumin concentrations. A difference of less than 1 g/dL (10 g/L) implies an exudate [5]. Nowadays, the serum-ascites albumin gradient (SAAG) is used to determine the cause of ascites [6,7]. SAAG≥1gr/dL (high gradient) indicates the ascites is due to portal hypertension, and SAAG≤1gr/dL (low gradient) indicates the cause of ascites is not associated with increased portal pressure [8].

The occurrence of postoperative adhesions is closely linked to several predisposing factors such as the type of treated organ, operation type, used materials, surgical manipulation, surgical complications, drainage tubes, and subjective reactivity [9]. Up to now, several therapeutic approaches have been evaluated to prevent or minimize the occurrence of adhesions; however, studies on ascites fluid have been limited. In this study, we aimed to evaluate the preventive effect of the ascites fluid for postoperative peritoneal adhesions in rat models.

## 2 Methods

The study is an experimental randomized controlled animal trail. Sixty Syrian male rat weightings 40±5gr were assigned to six groups of 10 animals each through randomization and in accordance with the guidelines of the animal ethics committee of Birjand University of Medical Sciences (Ref: IR. BUMS 1398.218). Sample size was calculated using the sample size formula for comparison of two means, at α = 0.05 and B (power) = 80%. Rats included in the study were all Syrian males, and in case of death or illness, were excluded from the study.

All surgical procedures were performed in the lab. The operator of the initial surgery was different from the evaluators of the re-laparotomy. Each rat was anesthetized with intraperitoneal ketamine hydrochloride (60 mg/kg) and xylazine hydrochloride (10 mg/kg). Before incision, the abdomen was shaved and prepared with povidone-iodine solution using a sterile technique. The abdominal cavity was opened via 2/5 cm vertical midline incision. Multiple measurable abrasions were made on the different sites of the peritoneal surface with a knife. Powdered gloves were also used during the procedures. Ascites fluid (2 mL) was poured into the peritoneal cavity of animals in the case groups (3 and 6). In the groups 2 and 5, 2 mL of normal saline was poured into the abdominal cavity of animals, and the abdominal wall was repaired in control groups 1 and 4 with no specific treatment. Then, the abdominal cavity in both groups was closed in a double layer with nylon 4-0 continuously. After that, the rats were housed in their standard plastic cages (2 rats per cage, 50✕50✕40 cm in size) under controlled temperature (21±2 °C), 58–65% humidity and a 12/12 light/dark cycles with food and water available. Due to intraperitoneal absorption and a decrease in intraperitoneal fluid by one third after 72 h, based on the pilot study, every three days 2 cc of the fluid of each group was injected into the peritoneum with a syringe.10 and 30 days after initial surgery the rats were killed and underwent another laparotomy in groups 1, 2, 3 and 4, 5, 6, respectively to assess macroscopic and microscopic adhesions, which were scored by a surgery resident who had no knowledge of the animals̕ group assignment. Macroscopic assessment was performed using an established scoring system which is explained in Table 1. This scoring system has been applied in several studies for scoring of the adhesion band [10] and evaluates the extent and severity of adhesions in the operation site. For microscopic assessment, we utilized the scoring system applied in the study of *Lashkarzadeh* et al. [11]. In this system, wound healing was graded in 3 categories as inflammation, proliferation and maturation phases clarified in Table 2. Each category has its own characteristics, and grading is based on some defined markers, as shown in Table 2. The site of the incision excised 10 and 30 days after initial surgery and preserved in formalin 10% concentration, then sent for wound healing grading by a pathologist. The pathologist was not aware of the groups each sample belonged to.

**Table 1:** Adhesion scoring system according to Canbaz and colleagues [12].

Degree of adhesion	Number of adhesion band
**0**	No adhesion
**1**	One adhesion band, no vessel, easily separated
**2**	Two thin adhesion bands, no vessel, easily separated
**3**	Three thin adhesion bands, no vessel, easily separated
**4**	More than three thin adhesion bands, easily separated with no vessel

**Table 2:** wound healing grading score [2].

Wound healing grade	Category
**1**	Inflammation markers: clot formation, PMN and macrophage infiltration, lack of collagen formation or new angiogenesis
**2**	Proliferation markers: fibroblast infiltration, collagen and proteoglycans synthesis, new angiogenesis, decreased of PMN cells and granulation tissue formation
**3**	Maturation markers: cellular and vascular depletion, scar formation

The ascites fluid was used in this study, prepared from one person with liver cirrhosis through abdominal paracentesis. The analysis of the applied ascitic fluid was described in Table 3.

**Table 3:** Analysis of the ascitic fluid.

Parameter	Level	Parameter	Level
Calcium (Ca)	6	Blood sugar (BS)	86
Amylase	21	Sodium (Na)	134
Lipase	34	Potassium (K)	3.6
Lactate dehydrogenase (LDH)	163	Albumin	0.9
PH	8.1	Protein	2.1
Blood Urea Nitrogen (BUN)	48	White Blood Cell (WBC)	0
Creatinine (Cr)	1.3	Red Blood Cell (RBC)	1–2

The work has been reported in accordance with the ARRIVE guidelines (Animals in Research: Reporting in Vivo Experiments) [13]. It has also been reported in line with Consolidated Standards of Reporting Trials (CONSORT) Guidelines.

Data was analyzed with descriptive statistics and analytical tests using SPSS v.18. Normality of data was checked with Kolmogorov-Smirnov test and data analysis was carried out with Kruskal Wallis and Bonferroni-corrected Mann-Whitney U tests. P-values of less than 0.05 were considered significant.

## 3 Results

Significant differences were found in distributing of the *macroscopic* adhesion frequency, 10 days after laparotomy, between the groups (P = 0.001), Table 4. The Bonferroni-corrected Mann-Whitney *U* test showed total macroscopic adhesion score was significantly lower in the ascites fluid treatment than in the control (P = 0.0001) or the normal saline (P < 0.001) group.

**Table 4:** Post-operative intra-abdominal adhesion band (*macroscopic adhesion*) between groups10 days after laparotomy.

Groups Grade	Control **Group f (%)**	**Normal Saline Group f (%)**	Ascites **Group f (%)**	Kruskal Wallis Test
**0**	0	0	1(10)	P = 0.001
**1**	0	1 (10)	6 (60)	
**2**	1 (10)	2 (20)	2 (20)	
**3**	6 (60)	3 (30)	1 (10)	
**4**	3 (30)	4 (40)	0	

Significant differences were also found in distributing of the *microscopic* adhesion frequency, 10 days after laparotomy, between the groups (P = 0.001), Table 5. The Bonferroni-corrected Mann-Whitney *U* test showed total microscopic adhesion score was significantly lower in the ascites fluid treatment than in the control (P = 0.0001) or the normal saline (P < 0.001) group.

**Table 5:** Distributing of the *microscopic* adhesion frequency 10 days after laparotomy between the groups.

Groups Grade	Control **Group f (%)**	**Normal Saline Group f (%)**	Ascites **Group f (%)**	Kruskal Wallis Test
**0**	0	1 (10)	7 (70)	P = 0.001
**1**	4 (40)	2 (20)	3 (20)	
**2**	4 (40)	4 (40)	0	
**3**	2 (20)	3 (30)	0	

Significant differences were found in distributing of the *macroscopic* adhesion frequency, 30 days after laparotomy, between the groups (P = 0.001), Table 6. The Bonferroni-corrected Mann-Whitney *U* test showed total macroscopic adhesion score was significantly lower in the ascites fluid treatment than in the control (P < 0.0001) or the normal saline (P = 0.001) group.

**Table 6:** Post-operative intra-abdominal adhesion band (*macroscopic adhesion*) between groups30 days after laparotomy.

Groups Grade	Control **Group f (%)**	**Normal Saline Group f (%)**	Ascites **Group f (%)**	Kruskal Wallis Test
**0**	0	0	0	P < 0.001
**1**	0	1 (10)	5 (50)	
**2**	0	2 (20)	2 (20)	
**3**	1 (10)	2 (20)	2 (20)	
**4**	9 (90)	5 (50)	1 (10)	

Significant differences were found in distributing of the *microscopic* adhesion frequency, 30 days after laparotomy, between the groups (P = 0.001), Table 7. The Bonferroni-corrected Mann-Whitney *U* test showed total microscopic adhesion score was significantly lower in the ascites fluid treatment than in the control (P < 0.0001) or the normal saline (P = 0.001) group.

**Table 7:** Distributing of the *microscopic* adhesion frequency 30 days after laparotomy between the groups.

Groups Grade	Control **Group f (%)**	**Normal Saline Group f (%)**	Ascites **Group f (%)**	Kruskal Wallis Test
**0**	0	1 (10)	9 (90)	P < 0.001
**1**	1 (10)	3 (30)	1 (10)	
**2**	7 (70)	5 (50)	0	
**3**	2 (20)	1 (10)	0	

There were no significant differences in terms of both macroscopic and microscopic adhesion intensity, 10 and 30 days after laparotomy between the groups (P > 0.05) Table 8.

**Table 8:** Macroscopic and microscopic adhesion intensity10 and 30 days after laparotomy in study groups.

Group		Macroscopic adhesion		Microscopic adhesion	
		Test Statistics	p- Value	Test Statistics	p- Value
Control 10	Control 30	−2.67	0.07	−1.07	0.314
Normal Saline 10	Normal Saline 30	0.28	0.781	−0.8	0.421
Ascites 10	Ascites 30	−1.11	0.261	−1.09	0.271

**Fig. 1:**
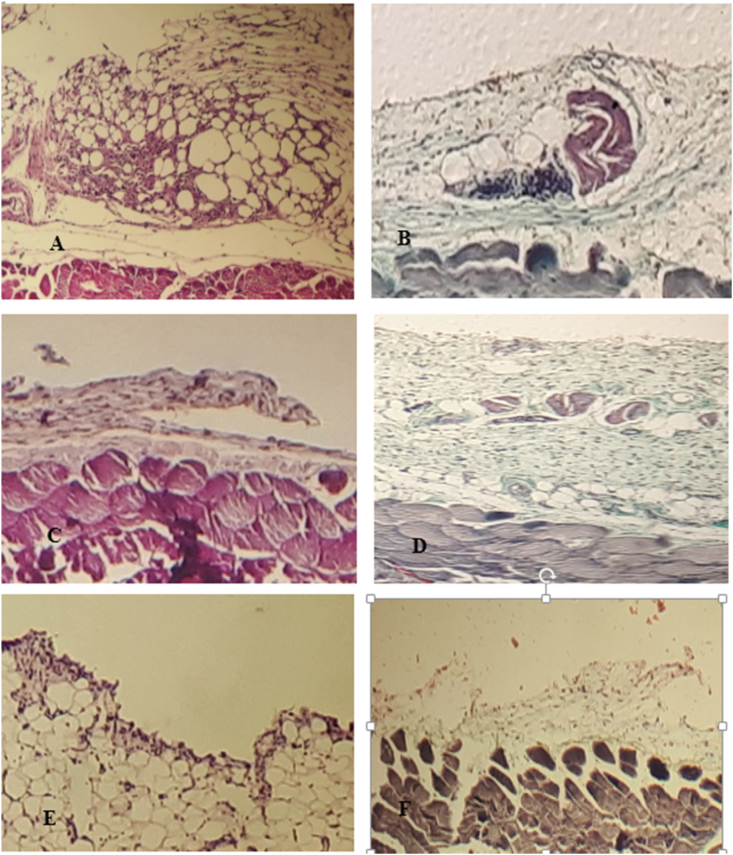
A: The histopathological view of microscopic adhesion 10 days after laparotomy in control group: Serosa with moderate to severe infiltration of lymphoplasmacells and few neutrophils with ectatic small vessels and mild intercellular edema B: The histopathological view of microscopic adhesion 30 days after laparotomy in control group: Serosa with moderate infiltration of lymphoplasmacells and few neutrophils with ectatic small vessels and few intercellular edema C: The histopathological view of microscopic adhesion 10 days after laparotomy in normal saline group: Serosa with mild to moderate infiltration of lymphoplasmacells and few neutrophils with ectatic small vessels and a few intercellular edema D: The histopathological view of microscopic adhesion 30 days after laparotomy in normal saline group: Serosa with mild to moderate infiltration of lymphoplasmacells and few neutrophils with ectatic small vessels and a few intercellular edema E: The histopathological view of microscopic adhesion 10 days after laparotomy in ascites group: Serosa with mild infiltration of lymphoplasmacells and few neutrophils with ectatic small vessels and a few intercellular edema F: The histopathological view of microscopic adhesion 30 days after laparotomy in ascites group: Serosa with mild to moderate infiltration of lymphoplasmacells and few neutrophils with ectatic small vessels and a few intercellular edema.

The histopathological views of microscopic adhesion 10 and 30 days after laparotomy in the different study groups are shown in Fig. 1.

**Fig. 2:**
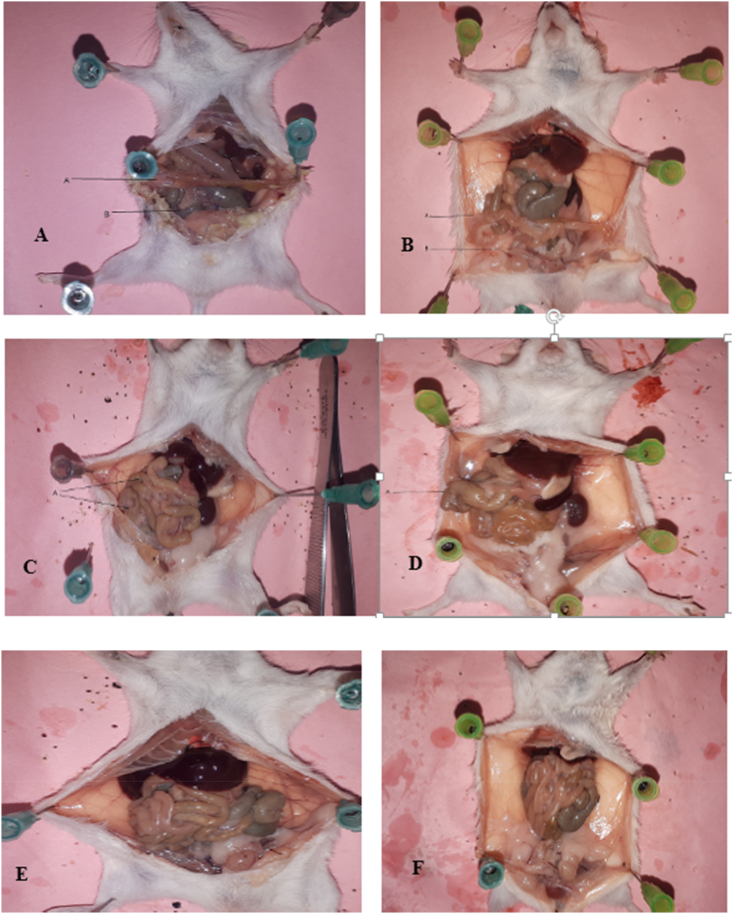
A: Intra-abdominal view10 days after laparotomy in the control group: 9 rats had 3 or more than 3 fibrous bands, a: Adhesion to the abdominal wall, b: Bonding adhesion between viscera B: Intra-abdominal view 30 days after laparotomy in the control group: 9 rats had more than 3 fibrous bands, a) Adhesion to the abdominal wall, b) Bonding adhesion between viscera C: Intra-abdominal view10 days after laparotomy in normal saline group: 7 rats had 3 or more than 3 fibrous bands, a) Bonding adhesion between viscera D: Intra-abdominal view 30 days after laparotomy in normal saline group: 7 rats had 3 or more than 3 fibrous band, a) Adhesion to the abdominal wall E: Intra-abdominal view10 days after laparotomy in ascites group: 1 rat had 3 fibrous bands, no more than 3 fibrous bands were seen, No adhesion F: Intra-abdominal view 30 days after laparotomy in ascites group: 3 rats had 3 or more than 3 fibrous bands.

The intra-abdominal views of rats in different study groups 10 and 30 days after laparotomy are shown in Fig. 2.

No adhesion.

## 4 Discussion

Our findings revealed that post-operative intra-abdominal adhesion was reduced in the ascites group, while no statistical differences were found in the intensity of adhesion, measured 10 vs. 30 days of examination. As indicated by these findings, ascites fluid can decrease post-operative intra-abdominal adhesions, which is in agreement with other findings [14]. As shown, the use of physiological liquids inside the abdomen decreases postoperative adhesions. Adhesion formation is a major concern of all surgeons. Different types of adhesion-reducing substances have been applied in animal models, but we are still far from the ideal substance.

Postoperative adhesions often develop as a result of peritoneal injury, cell death, and blood remnants, resulting in fibrin deposition [15]. Surgical methods to diminish postoperative adhesion formation involve minimal tissue handling and reduced peritoneal trauma [16]. Although these strategies are helpful, they do not completely prevent the incidence of adhesion. Therefore, the advancement of adhesion preventive agents or devices is essential [16,17].

We used natural material, ascites fluid, to prevent adhesion formation. The peritoneum is a serous membrane that serves to support the organs of the abdomen and acts as a conduit for the passage of nerves, blood vessels, and lymphatics. In the steady state, approximately 20%–40% of the fluid that flows into the tissue of the peritoneal cavity is absorbed by interstitial lymphatics, and that approximately 60%–80% of the fluid is absorbed by blood capillaries. One hypothetical mechanism of action is that, Injection of the ascites fluid may dilute the products of the inflammatory processes and transmit them to the lymph. Performing the biochemical examination for CRP or cytokine markers is necessary to confirm this hypothesis in future studies. The following mechanisms are involved in preventing the formation of the fibrous band: a) prevention of coagulation of serous secretions, b) dissolve the fibrin formed at the tissue surface, and c) mechanical barriers between injured peritoneal surfaces. Numerous studies have been performed on adhesion formation that the mechanism of all of them was mechanical barriers between injured peritoneal surfaces [10,[18], [19], [20], [21]].

Barriers application sometimes make the treatment difficult. Elyasi et al. (2017) showed that the use of herbal substances was an equally effective method in the prevention of peritoneal adhesion [22]. Substances are easy to apply and have comparable efficacy in adhesion prevention as other barriers. Similarly, the results of a study by Tahmasebi et al. revealed amniotic fluid can decrease the likelihood of post-operative intraperitoneal adhesion formation (10). Based on our clinical experience, people with ascites in case of surgery had less adhesion formation than others.

Our study, in a rat model of post-surgical adhesion formation and prevention, approaches adhesion prevention from a new perspective that aimed to test whether the ascites fluid from a human with liver cirrhosis can decrease the rate and severity of post-operative adhesions. One limitation of the study is that we do not know exactly which component or components of ascites fluid reduce the strength or number of the adhesion bands. Longer follow-up and further histological evaluation of tissue samples are required. Further studies on the application of low SAAG ascites fluid are also recommended.

## 5 Conclusion

This experimental study investigated the prevention of post-operative adhesion formation by ascites fluid. Ascites fluid can decrease the possibility of post-operative intra-peritoneal adhesion formation. Additional studies will be needed to ascertain whether ascites fluid is effective in long-term adhesion reformation. Finding an effective agent to decrease adhesion formation would improve the post-operative course for surgical procedures with a high risk of adhesion formation.

## Availability of data and materials statement

The datasets used and/or analyzed during the current study are available from the corresponding author on reasonable request.

## Provenance and peer review

Not commissioned, externally peer-reviewed.

## Ethical approval

The animal ethics committee of Birjand University of Medical Sciences (Ref: IR. BUMS 1398.218).

## Sources of funding

Birjand University of Medical Sciences.

## Author contribution

Ali Shaykh o Islami and Mohammadreza Ghasemian**:** Study design and calculating, Marjan Farzad: Writing and editing, Mahmoud Zardast**:** Pathology Report.

## Research registration Unique Identifying number (UIN)

• Name of the registry: N/A
• Unique Identifying number or registration ID: N/A
• Hyperlink to your specific registration (must be publicly accessible and will be checked): N/A

## Guarantor

Ali Shaykh o Islami and Mohammadreza Ghasemian.

## Declaration of competing interest

The authors declare no competing interest.
